# Early Detection of Oral Potentially Malignant Disorders: A Review on Prospective Screening Methods with Regard to Global Challenges

**DOI:** 10.1007/s12663-022-01710-9

**Published:** 2022-04-15

**Authors:** Neda Haj-Hosseini, Joakim Lindblad, Bengt Hasséus, Vinay Vijaya Kumar, Narayana Subramaniam, Jan-Michaél Hirsch

**Affiliations:** 1 Department of Biomedical Engineering, Linköping University, Linköping, Sweden; 2 Centre for Medical Image Science and Visualization, Linköping University, Linköping, Sweden; 3 Centre for Image Analysis, Department of Information Technology, Uppsala University, Uppsala, Sweden; 4 Department of Oral Medicine and Pathology, Institute of Odontology, University of Gothenburg, The Sahlgrenska Academy, Gothenburg, Sweden; 5 Clinic of Oral Medicine, Public Dental Service, Gothenburg, Region Västra Götaland Sweden; 6 Department of Head and Neck Oncology, Sri Shankara Cancer Hospital and Research Centre, Bangalore, India; 7 Department of Surgical Sciences, Odontology and Maxillofacial Surgery, Medical Faculty, Uppsala University, Uppsala, Sweden; 8 Department of Research & Development, Public Dental Services Region Stockholm, Stockholm, Sweden

**Keywords:** Artificial intelligence, Assisted screening, Noninvasive methods, Oral cancer, Optical imaging

## Abstract

Oral cancer is a cancer type that is widely prevalent in low-and middle-income countries with a high mortality rate, and poor quality of life for patients after treatment. Early treatment of cancer increases patient survival, improves quality of life and results in less morbidity and a better prognosis. To reach this goal, early detection of malignancies using technologies that can be used in remote and low resource areas is desirable. Such technologies should be affordable, accurate, and easy to use and interpret. This review surveys different technologies that have the potentials of implementation in primary health and general dental practice, considering global perspectives and with a focus on the population in India, where oral cancer is highly prevalent. The technologies reviewed include both sample-based methods, such as saliva and blood analysis and brush biopsy, and more direct screening of the oral cavity including fluorescence, Raman techniques, and optical coherence tomography. Digitalisation, followed by automated artificial intelligence based analysis, are key elements in facilitating wide access to these technologies, to non-specialist personnel and in rural areas, increasing quality and objectivity of the analysis while simultaneously reducing the labour and need for highly trained specialists.

## Introduction

### The Problem

Cancer in the oral cavity including lips has an age standardised incidence rate of 4.1 per 100,000 worldwide for both genders and all ages, as reported in 2020 by the World Health Organization (WHO). Oral cancer is a serious public health problem in India in particular; 135,000 new cases and 75,000 deaths were reported in 2020, which is approximately one-third of the global incidences and deaths due to this cancer [2]. One of the reasons oral cancer remains endemic in the region is the high incidence of tobacco use; almost a third of adults in India use tobacco as per the last Global Adult Tobacco Survey [3]. Despite evidence that screening for oral cancer significantly reduces mortality in high-risk groups such as tobacco users and individuals with excessive alcohol consumption, organized screening efforts are absent in most parts of India.

In India, on average, there is a delay of nine months from the onset of symptoms to diagnosis. Of this, seven months are attributed to delays within the medical pathway [4]. A primary reason is the need for biopsy to confirm the diagnosis. In order to perform evaluation and biopsy, a patient with suspicion of oral cancer in a tier-two or tier-three city is often referred to a larger centre which may be hundreds of miles away. Additionally, the biopsy requires, on average, a day and half for a report; hence, patients of suspected oral cancer need to undergo significant hardship to establish a diagnosis. Most of the population lives in a rural environment, while most healthcare establishments are concentrated in urban areas—meaning these patients often do not have access to pathology services and expertise. Without officially confirmed proof of cancer, they are not eligible for state-run insurance schemes for treatment. A delayed diagnosis increases the cost of treatment, and it drastically reduces likelihood of survival. In India, the higher incidence of oral cancer and often advanced stage at diagnosis leads to an expenditure of 386 million USD each year due to the productivity loss only for this cancer type, which is considerably higher than the comparable demographic and economic growing countries [5].

Several studies indicate that there is a link between the degrees of dysplasia and malignant cell transformation, while others claim that there is no such link [6, 7]. Systematic reviews of retrospective data report rates of 0.1% to 40% of oral leukoplakias (OL) that develop into oral cancer [8, 9], indicating inconsistency in the studies as well as dysplasia grading for risk assessment of progression to cancer [10, 11]. In a well-defined cohort, Jäwert et al. report on a 11.5% risk of OL transformation to oral squamous cell carcinoma (OSCC) during a median follow-up of 9 years with an annual transformation of 3.5%, where non-homogeneous OL with dysplasia located on the tongue showed a significantly higher risk than other locations. However, the factors that control this progression are still unclear, creating significant difficulties in providing predictions and indicating the need for continuous patient monitoring.

The 5-year overall survival rate in developed countries in all the stages combined ranges from 30% to 80% [2, 12]. Those with early stages (1 and 2) have a better 5-year survival probability, whereas patients with advanced stages have a lower survival rate (74% vs. 36%) [12]. The type of treatment also has an impact on survival as evidenced in older studies, where more patients treated with radiotherapy. Surgery followed by radiotherapy has become standard treatment for oral cancer patients, especially in advanced stages [6]. The recurrence rate after total excision of an OL is approximately 50% after five years [13]. In general, quality of life for patients after oral cancer treatment is severely hampered [14].

### The Solution

*‘Early detection and early treatment’* is one of the main solutions for preventing progression of the disease, improving patient quality of life, survival and to reduce morbidity. Several studies point out the benefits of screening for oral cancer. In a fifteen-year study in Kerala, India, trained health workers visually examined the oral mucosa of tobacco and possible alcohol consumers every third year, which resulted in a sustained reduction in oral cancer mortality. The largest reduction was noted in the group that adhered to repeated visual screening sessions. Patients with suspected potentially malignant oral disorders (PMOD) or cancer were referred to specialist diagnostic clinics, as assessment of PMOD is challenging by visual examination alone. Similarly, Rethman et al. found that visual screening can reduce mortality in oral cancer, especially in a group of tobacco users with or without concomitant alcohol consumption [15]. To ensure a diagnosis, tissue biopsies are required, especially in the event of changes in several different sites [16].

This review explores the different methods, sample-based and direct imaging of the patient, that are available as a complement to clinical examination and tissue biopsy for early detection of PMOD. We also discuss the analysis methods that facilitate interpretation of the images or data for the clinicians.

## Methods

The methods for acquiring the diagnostically relevant information can roughly be grouped in two types, they are either based on direct observation or imaging of the patient (in vivo), or they are based on analysis of extracted material from the patient (ex vivo). The former both suffer and benefit from being closely tied to the patient. Direct imaging of the oral cavity of the patient requires that the necessary imaging devices are available at the point of care, thereby ruling out expensive devices or such which require highly skilled personnel. On the other hand, the immediate availability of the patient allows increased flexibility to adjust imaging to the situation which simplifies the work and provides fast feedback. The methods which rely on extracted samples may, if resources are available, be analysed at the point of care, but such approaches also provide options where the sample is transported to a nearby laboratory for analysis. This may enable use of the same resources to cover a larger region, thereby allowing cost-efficient use of more expensive equipment. The sample-based approach may also allow self-sampling, which could be a suitable option for saliva and brush samples.

### Sample-Based Methods

Methods based on imaging of samples (ex vivo) avoid the need for local access to instrumentation and staff to handle it, since samples can be transported to where technical resources and expertise are available. This is an important advantage for being able to offer equivalent health care at a reasonable cost also in less accessible rural areas.

#### Histology Using Tissue Biopsies

**Fig. 1:**
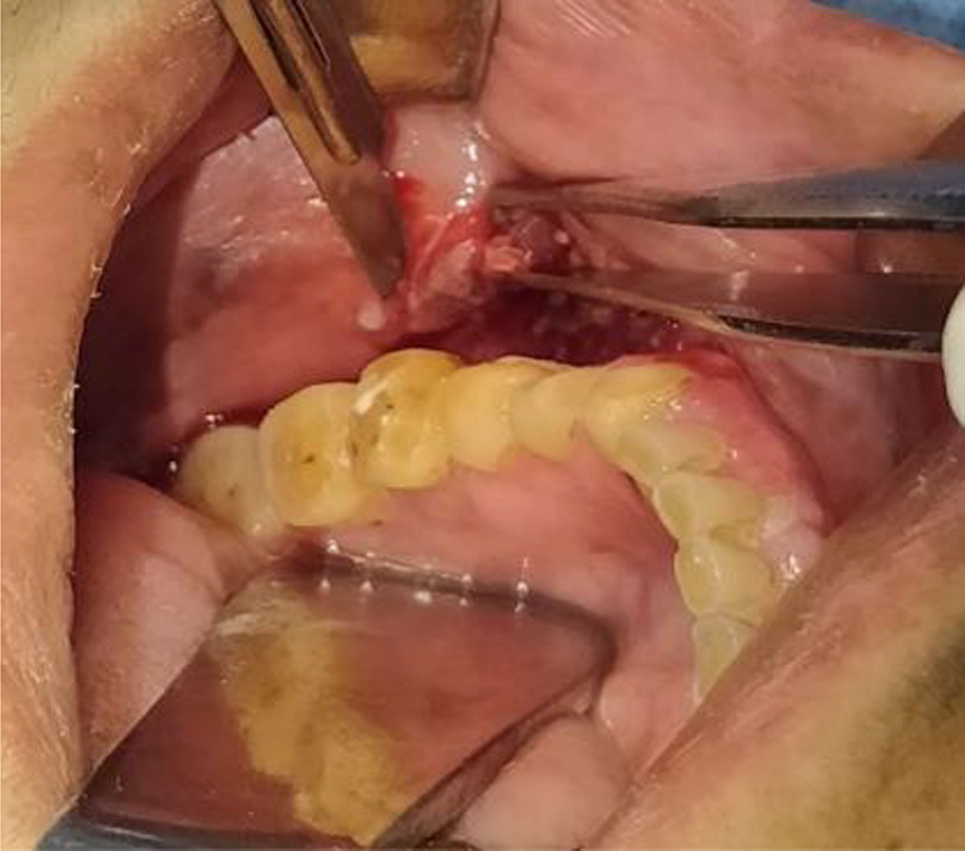
Incision biopsy

Histological analysis of tissue biopsy is the current ‘gold standard’ for clinical examination, it is based on tissue biopsy, slide preparation, histological staining and microscopic analysis (manual or computer assisted). Tissue specimens can be obtained by scalpel or a punch, depending on the appearance of the tissue or preference of the treating dentist/surgeon. Where technically possible, complete excision of diseased tissue is preferable. Otherwise, a ‘mapping procedure’ may be used to mark where incisional biopsies (Fig. 1) are obtained in areas with different reaction patterns. It is of importance that representative biopsies are obtained: that they are sufficiently large to include normal and suspicious tissue and the full depth of the mucosa, in order for the pathologist to give a diagnosis without requiring further samples. Tissue specimens should be submitted, in buffered formalin to ensure proper fixation, to a pathology laboratory for slicing, staining, and histopathological diagnosis. Based on the report, it is decided when the patient should be reviewed at the specialist dental care, considering the risk of tumour development. This may require repeated biopsies and may be warranted if a change in the clinical picture occurs. The value of histologic assessment is unclear because the description and grading of dysplasia are subjective [10]. However, the general opinion is that assessment of PMOD by clinical examination alone is challenging and in order to confirm a clinical diagnosis, tissue biopsy is required, especially in the event of lesions in multiple locations.

#### Cytology Using Brush Biopsies

**Fig. 2:**
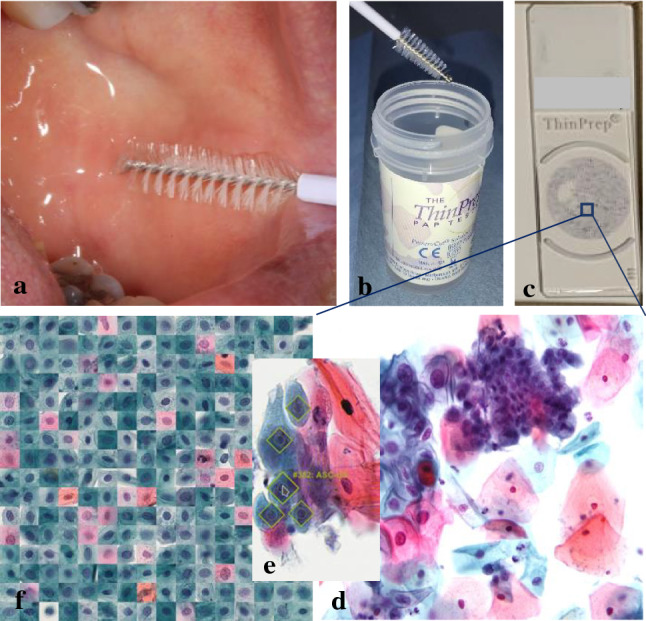
Liquid based cytology process cycle: **a** sampling with a brush, **b** deposition into a preservative liquid, **c** whole slide image cytology, **d** close-up of the cells, **e** extraction and annotation of the cells for AI analysis, and **f** mosaic of the extracted cells

Cytological analysis relies on microscopic examination of collected cells that have been spread out on a glass slide and stained. Cell samples are taken by a brush from PMOD or changes that are suspected to be cancer (Fig. 2). Samples are either smeared directly onto the glass or deposited into a small bottle of preservative liquid which is later spread onto the glass. Staining is most often performed according to Papanicolaou, which is a polychromatic substance involving multiple dyes that stain different components of the cell with different colours and intensities [17]. Currently, the consensus is that liquid-based cytology (LBC) provides an improvement on specimen adequacy, visualisation of cell morphology and diagnostic reproducibility [18]. LBC also simplifies cell collection due to easier handling and fewer transfer errors. A cytotechnologist, aided by a microscope, looks for any cells showing signs of malignant changes among approximately 100,000 cells in a sample, marking cells of interest. This process takes 10–15 min. As a following procedure, a cytopathologist analyses the samples and the indicated areas of interest to set the diagnosis.

The Cochrane meta study, summarising results from 20 oral cytology studies, reports sensitivity of 90% and specificity of 94% for the detection of oral cancer and PMOD [19]. Deurling et al. analysed and compared 1352 brush biopsies taken for cytological diagnosis with the same number of pathological-anatomical analyses of tissue biopsies of oral lesions. The results showed that LBC based on brush biopsies has high sensitivity (95%) and specificity (85%) and the authors concluded that brush samples are fully reliable for the diagnosis of neoplasia in the oral cavity [20]. Thus, with current technology, brush biopsies appear to be a reliable alternative to tissue biopsies for diagnosing cell changes [21].

No local anaesthesia or suturing are needed for the brush biopsy and acquiring the sample is relatively easy, in contrast to a tissue biopsy. Brush biopsies are less expensive, require less resources and are considerably less invasive for the patient. In ongoing studies, dental hygienists and dentists are being evaluated as performers of brush tests in general dental practice (GDP). Preliminary results show that 98% of acquired brush biopsies can be used for diagnostics [22, 23]. Accordingly, sampling could routinely be done in GDP and possibly also as self-sampling, similar to screening for cancer of the cervix, which has been successful [24].

#### Liquid Biopsies

##### Saliva-Based Biomarkers

Saliva contains various biomarkers that signal disease, and it has great potential for early cancer diagnosis with cost-effective and easy collection, storage, transport and processing. A systematic literature review [25] has evaluated studies regarding possible saliva markers for PMOD and oral cancer in both unstimulated and stimulated morning saliva. These studies showed that most of the possible markers are proteins. The authors’ conclusion was that a combination of biomarkers in saliva could be used as a screening tool to improve early detection and diagnostic safety of PMOD and cancer. However, methods of saliva collection, processing, storage methods and analysis must be standardised, prior to clinical implementation. Likewise, limit values for different salivary biomarkers must be defined for healthy individuals, and for individuals with PMOD or oral cancer. Additionally, metabolites are differentially expressed in saliva of subjects with oral cancer as compared to normal subjects [26]. The best possibility for the development of diagnosis using saliva-based biomarkers, should be based on a combination of biomarker panels after standardisation of the procedure, which could then be used as an effective screening tool to improve early detection and diagnostics [26]. The use of saliva-based biomarkers for cancer detection is still in early development, and no study has yet reported on the diagnostic accuracy of salivary sample analysis according to the Cochrane review [19].

##### Blood Samples

Circulating tumour DNA (ctDNA) are small pieces of DNA found in the bloodstream, originating from cancerous cells that have died. ctDNA can be differentiated from normal cell DNA by the presence of several alterations. Results so far suggest that ctDNA can detect cancer in close to 70% of the head and neck cancer cases, while the performance varies significantly, correlating with tumour type among all cancers and stages (between 8 and 100%). The larger the size and spread of the tumour, the higher the sensitivity of the method. A proof of concept in a prospective randomised screening trial shows that a better prognosis for patients diagnosed using ctDNA is still to be presented [27, 28].

### Assisted Screening of the Oral Cavity

As an alternative or complement to sample-based diagnosis, the assisted screening of the oral cavity of the patient can potentially provide a quick, painless, and affordable examination that can be integrated into any routine examination. Among the various techniques, optical methods are easy to handle, safe, and already implemented within dentistry. In this section, fluorescence, Raman techniques and optical coherence tomography (OCT) are considered as methods with the highest potential for the purpose of this review.

#### Direct Inspection Under White Light

The initial and conventional oral examination is the visual inspection and palpation of the oral cavity by a specialist, based on WHO’s oral cancer diagnosis protocol [29]. This method allows direct feedback to the patient. Unfortunately, the reliability of such analysis is low, subjective, and requires skilled personnel. An alternative, which makes the optical analysis more objective, is to take digital white light photographs of the oral cavity. This allows remote human analysis, as well as computer-supported analysis. To further improve the contrast, different types of staining may be used, e.g. toluidine blue [30]. Light-based methods, including white-light and autofluorescence (AF), have shown a sensitivity of 87% and specificity of 50% for the detection of PMOD and oral cancer in meta-analysis of 23 studies [19].

#### Fluorescence Techniques

Tissue is composed of naturally fluorescing molecules (fluorophores) that, when excited with light in their specific absorption spectrum, emit a spectrum of light with longer wavelengths referred to as AF. Ultraviolet-violet light is commonly used as excitation light with fluorescence in the visible range that can be directly observed by the human eye. Pathological changes in the tissue lead to alterations in the tissue fluorophores and thereby AF, where cancerous tissue often emits AF with a lower intensity. The main contributors to autofluorescence in normal oral tissue are known to be epithelial fluorescence emitted from metabolic molecules originating from the cell cytoplasm, and stromal fluorescence originating from collagen [31]. Changes seen in AF are reported to be associated with loss of collagen in the stroma and the increased metabolic activity of the cells. In cancer, changes in the cells in the stroma precede changes in epithelial cells and invading tumours [31, 32].

**Fig. 3:**
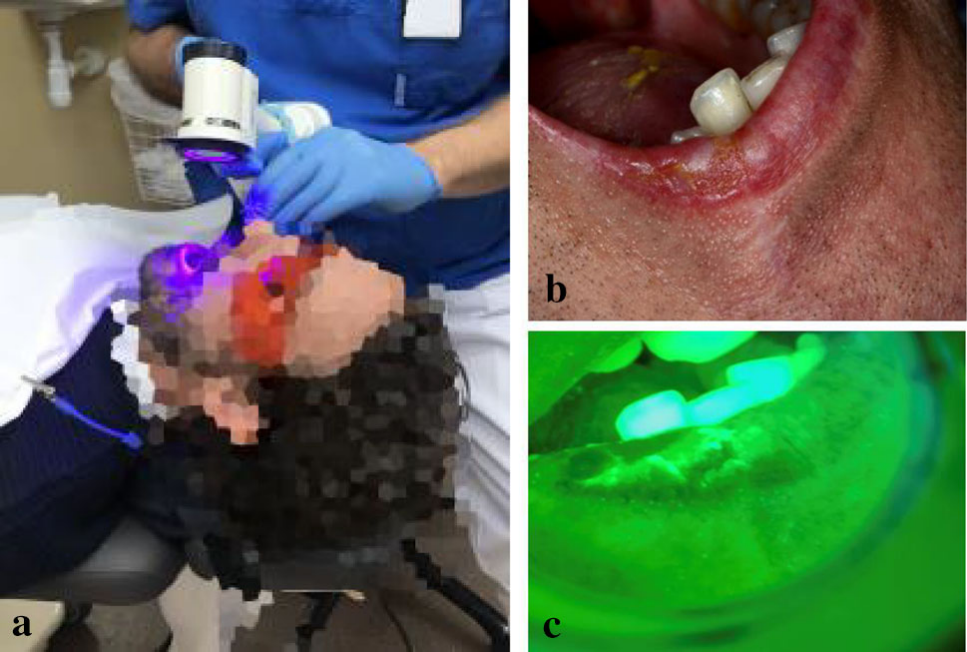
**a** Assisted screening using VELscope^®^ used with safety goggles, **b** white light image of epithelial cancer in the right lip, and **c** fluorescence image of the same site. The person in (**a**) is not a real patient. **b** and **c** are re-published with permission from Tandläkartidningen [1]

The main advantages of AF-based examination methods are that no substance needs to be administered to the patient, no fixation or staining of samples is required, and the measurement techniques are relatively simple and inexpensive. Tiwari et al. and Lima et al. [33, 34] have reviewed the efficacy of clinically evaluated fluorescence imaging methods. The studies report different sensitivity and specificity values for AF imaging; however, they all show a higher sensitivity by combining AF imaging with conventional oral examination. Different commercial or research-grade devices have been implemented for visualisation of AF, where VELscope^®^, a handheld instrument for direct visualisation of AF using a blue excitation light, was used more extensively than other devices. Through the VELscope^®^, malignant tissue shows darker (no or low AF emission) compared to the surrounding healthy tissue that emits green AF (Fig. 3). However, it is reported that the excitation light and the pathology and anatomic site of the lesions affect the total AF [31] which is not considered in most of the clinical studies and is a probable reason for the variation in reported efficacies. Direct visualisation of AF is reported not to have a high diagnostic performance for distinguishing dysplasia or early cancer, which questions its reliability, especially in GDP, where experience with the system is usually lacking. Still, the WHO considers VELscope^®^ to be an effective tool for the prevention of oral cancer [35].

With the addition of photosensitizing agents, i.e. exogenous fluorescent markers, the contrast of the target cancer tissue is enhanced, giving more reliable signals or images compared to AF. Among the considered agents, 5-aminolevulinic acid (5-ALA) is the most investigated substance for oral cancer, with the possibility of being topically applied to the lesion [34, 36]. 5-ALA is yet not approved for oral cancer detection; however, initial studies show a high sensitivity but a lower specificity (90–100% vs. 60–90%) [34] and dependence of the results on the anatomic site [37]. The use of either spectroscopy methods together with spectral analysis or advanced quantitative imaging could be beneficial for visualising both the AF and the exogenous marker’s fluorescence.

#### Raman Techniques

Information on the chemical composition of tissue can be extracted by measuring the vibrational modes of the molecules in the Raman scattered light spectrum. The method is label-free and specific, but the signals are weak, and interpretation of the optical spectra requires extensive knowledge and experience of the method, making the extraction of information highly dependent on the analysis and statistical methods. However, the interest for implementing the technology clinically and for surgical applications is rising, leading to adaptation of the technology for such circumstances [38, 39]. Systems used in the clinics are mostly spectroscopic, endoscopic [40] or in the form of a handheld imager [41]. Studies show promising results for oral cancer detection; however, the results are reported differently based on the measurement systems used and molecular content detected. A high sensitivity has been achieved for discrimination of oral cancer from normal tissue distinguished mainly at the protein, amino acid level, and with beta-carotene as the main molecular markers [42]. A clear distinction of OSCC and normal tissue is seen as an increase in protein and DNA, and a decrease in lipid content [43]. Difference in the water content of tissue has also shown to be useful for distinguishing tumour margins during oral cancer surgery [44]. Moreover, Raman imaging microscopy has shown positive results for grading of chondrogenic tumours based on the tissue histology slides [45]. However, Raman systems are currently relatively expensive for implementation in small clinics.

#### Optical Coherence Tomography

OCT is a method based on low-coherence interferometry that scans 2D images from which 3D images can be reconstructed. OCT is suitable for imaging of the tissue microstructure with micrometre resolution and allows an imaging depth of about 1–3 mm in the tissue. Application of OCT is well established within routine ophthalmology and cardiovascular examination. The potentials are numerous for other clinical imaging and screening applications as well, including oral cancer screening. There are, however, several obstacles for expansion of the OCT application to a routine screening: the system costs, the need for careful preclinical studies, non-trivial interpretation of the images and relatively bulky systems. As the technology develops, handier systems at affordable costs will become available, making OCT a suitable option for routine screening of oral lesions.

**Fig. 4:**
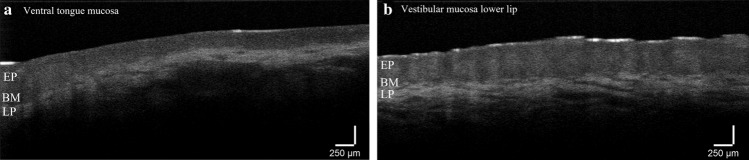
OCT image of **a** ventral tongue mucosa and **b** vestibular mucosa in the lower lip. The images illustrate approximately 0.8 mm deep in the tissue with layers of epithelium (EP), basement membrane (BM) and lamina propia (LP). The layer thicknesses are clearly different between **a** and **b**

A sufficient number of studies have reported on the capability of OCT to distinguish between normal, dysplasia and malignant oral tissue [46, 47], suggesting that the method has potential for this application. Smith et al. [48] could with sensitivity and specificity > 93% distinguish cancer vs non-cancer (normal and dysplasia). Polarisation-sensitive OCT imaging (PS-OCT) has shown a higher image quality and resolution [49, 50] and very high accuracy in distinguishing benign from malignant (dysplasia or early cancer) oral lesions in mice [49]. Disregarding the image quality and details apparent in the image, interpretation of the images is often not trivial and therefore appropriate image analysis and classification methods are beneficial for facilitating the implementation of OCT in the clinic. Examples of OCT images from the oral mucosa obtained using a non-PS spectral domain system are shown in Fig. 4.

## Digital Data Analysis

To reduce cost, labour and subjectivity, and to increase accuracy and speed, computer aided analysis of the information acquired is highly desirable. Digital analysis can be performed together with all the above-mentioned techniques. Additionally, with the recent immense progress in artificial intelligence (AI) and in particular deep learning (DL), the learning-based analysis of data has rapidly become a powerful and important part of medicine. Combining this with the growing global shortage of trained physicians, it is evident that digitalisation of the data and its analysis is essential for larger-scale efforts and screening initiatives. A digital format of data may be an integral part of the technology, such is the case for, e.g. OCT and Raman techniques, while for others, this requires an additional step, often in the form of digital imaging, such as in histology and cytology.

### Digital Pathology

With the progress in digital techniques such as scanning microscopy and automatic analysis of digital images in histology and cytology, we observe that digital utilities are playing an increasingly dominating role as an adjunct to ordinary visual microscopy. To establish routine solutions for primary diagnosis from a digital slide, so-called whole slide imaging (WSI), where a high-resolution image of an entire glass slide at a specific magnification is produced, is growing in importance. The work process is then to interact with digital slides on a computer monitor in a similar way to viewing a glass slide in a light microscope [51]. While rapidly becoming the standard in histology, the implementation of WSI is slower for cytology. This is partly due to higher requirements on the imaging; while a single focal plane suffices for histological analysis, the thicker, intrinsically three-dimensional, cytology samples generally require multiple focal planes to capture adequate diagnostic information. At present, there are only a limited number of studies available for oral pathology [52, 53], but the number is increasing and there is strong evidence of the usefulness of WSI systems for utilisation in primary diagnosis.

The digital slides can be transmitted quickly, regardless of geographic limitations and without risk of losing or damaging slides. Therefore, digital pathology is useful for remote diagnosis, workload balance, teleconsultation, quality control, image analysis and research, and will most likely be the workhorse in the future, replacing today's glass slides. However, there are obstacles such as high instrumentation costs and regular maintenance, a need for additional training, changes in traditional workflow, and integration with present software, and also the need for approval of the authorities to use the WSI system for primary diagnosis followed by validation in each clinical laboratory [54]. To gain the benefits of remote data access, there is a need for additional resources such as cloud servers, to store, manage and process the data, instead of a local server. This is important especially when multimodal big-data analysis is applied, where multiple sources of information, in addition to WSI and meta-data, are used. On the other hand, availability of big data supports the use of advanced computational techniques, in particular machine and deep learning, enabling high quality automated analysis which presents immense opportunities in healthcare.

### Automated Data Analysis

Although having access to the images in digital format facilitates remote access and improved quality control, the sheer workload of manual analysis is a major bottleneck which prevents large scale screening initiatives; due to the very large amount of data associated with large scale oral cancer screening [55], efficient and reliable computer-assisted examination is essential for the feasibility of a screening project. A vast range of mathematical, statistical, signal and image analysis methods can be implemented on the various types of data acquired. Digital fractal dimensions analysis (FDA) is an example of a model-based method that can estimate the risk of lesions using high-resolution digital photos, e.g. on white light images before and after staining with toluidine blue [56].

Thanks to the ongoing AI revolution, propelled by the outstanding performance and versatility of Deep Convolutional Neural Networks (DCNNs), image analysis tasks which some years ago were considered unsolvable can nowadays be addressed with standard tools. A complete DL-based system for detection of oral cancer in digital cytology is presented by Lu et al. [57]. Evaluation of several datasets in this pilot study confirms that the method can significantly reduce human burden while making the analysis more accurate; on a limited data set (18 patients), 100% patient level accuracy is reached. A benefit of learning-based systems is that with more data, performance can be expected to improve. Scaling up the system through additional training data and adjusting to increased sample variability, will with no doubt lead to further improvements in performance.

### The Potential for AI in Healthcare

Although the potential impact of AI in healthcare and life sciences is extraordinary, its actual usage is still very limited [58]. This is due largely to the fact that DL methods, although excellent at finding associations within the training data, are unable to distinguish what is causally relevant from what is accidentally correlated, such as artefacts of an imaging device. To reach successful integration and usage of AI systems in healthcare, an important step is to increase trust in the AI-based solution and improve human–machine collaboration in the decision-making process. The field of Explainable AI (XAI), which offers tools to enable interpretability and explainability of deep networks, has recently gained increased attention. XAI techniques allow visualising what information is used by the network in the decision-making. This enables monitoring of the process for increased reliability, but also presents possibilities to make new discoveries about the disease. Whereas methods to visualize where a network focuses its attention are fairly well developed [59], information on what the network is looking at in that particular location is still very limited. Understanding the ‘reasoning’ behind a decision of a deep learning model is particularly important [60], in order to confront the tendency of such models to appear highly susceptible to adversarial examples, outputting confidence scores over 99.99% for samples that resemble pure noise [61]. By integrating methods for XAI directly in the interface [62], it may be possible to offer an interactive tool for the best possible diagnostics at a minimal cost [63].

## Summary and Discussion

The goal of this paper is to review the methods and techniques that can contribute to reducing the prevalence of oral cancer, a mutilating disease with a very poor prognosis [64]. Clinical inspection alone can detect fulminant oral cancer but has limited capability in discriminating oral mucosal lesions and assessing the cellular changes in PMOD. The traditional way to determine risk is based on histopathology, where the severity of the lesion is graded. This procedure correlates well with the prognosis [65]. Since the recurrence rate of OL after total excision is relatively high [13], patients with a history of OL must be subjected to continuous reviews. Performing such monitoring by using of tissue biopsy and histopathological analysis is manageable in a privileged society with access to specialist care and costs partly covered by insurance systems, but it is beyond reach for the majority worldwide. Without a viable alternative, with often late reviews in most parts of the globe, and with no access to screening at regular visits to health centres or dental clinics, it is obvious that there is a need for adjunctive methods. These alternatives must be practical, low cost, easy to handle at a health centre and in GDP by a reasonably trained workforce to enable regular follow-up of asymptomatic patients with PMOD. These methods should have the potential to identify dysplastic or malignant lesions noninvasively, avoiding tissue biopsies that per se can drive malignant transformation [66]. It is clear from this review that there are several available potential alternatives. However, many of these adjunctive aids do not provide enough accuracy for early detection of cellular changes [65], or do not meet the requirements of simplicity and low costs. We primarily identify fluorescence imaging for oral cancer detection and oral brush sampling for cytology as the two most promising approaches in the near future, whereas OCT and Raman techniques, with further technological development, could offer suitable options in a longer perspective.

Most of the techniques require specialists for diagnostics, which is costly and an obstacle if introduction is to be on a large scale. Here, AI-based technologies open opportunities to radically reduce analysis costs, speed up management, and at the same time increase diagnostic accuracy. The integration of explanatory AI methods with a web-based interface appears to be a favourable path towards reaching the best possible diagnostics at a minimal cost [62, 63].

## Conclusion

There are two important factors to consider regarding technologies for early detection of potentially malignant diseases in the context of screening. Firstly, oral cancer is a global health issue and the population affected is diverse; it is therefore unlikely that a one-size-fits-all approach will be relevant. The most pragmatic approach will depend on the extent of the population to be screened, the availability of specialized equipment and trained personnel, as well as existing healthcare infrastructure. The second consideration is that, although many of these technologies are not yet mature for day-to-day clinical use, they all have a potential role to play in screening, which is meant to be fast, efficient, inexpensive, accurate and have a low turnaround time. A need for further studies, particularly towards validation and AI application, is observed in order to evolve a robust screening system which effectively aids early detection and results in better treatment outcomes. With effort invested in further development and validation of non-invasive techniques, combined with the growing potential of AI-supported analysis, conditions are present for creating a robust screening system which, based on noninvasive explainable techniques, can facilitate early detection of oral potentially malignant disorders and result in significantly improved treatment outcomes that can be applied globally.
